# Body Mass Index, Vitamin D, and Type 2 Diabetes: A Systematic Review and Meta-Analysis

**DOI:** 10.3390/nu10091182

**Published:** 2018-08-28

**Authors:** Shamaila Rafiq, Per Bendix Jeppesen

**Affiliations:** Department of Clinical Medicine Aarhus University, 8200 Aarhus, Denmark; shamailarafiq@clin.au.dk

**Keywords:** hypovitaminosis D, type 2 diabetes, body mass index, Vitamin D, BMI

## Abstract

The deficiency of vitamin D is prevalent all over the world. Studies have shown that vitamin D may play an important role in the development of obesity. The current study was conducted to quantitatively evaluate the association between serum 25-(OH) vitamin D levels and the risk of obesity in both diabetic and non-diabetic subjects. A systematic review and meta-analysis of observational studies was carried out for that purpose. We searched the Medline, PubMed, and Embase databases throughout all of March 2018. A total of fifty five observational studies for both diabetic and non-diabetic subjects were finally included in the meta-analysis. The data were analyzed by comprehensive meta-analysis software version 3 and the random effects model was used to analyze the data. The meta-analysis showed an overall inverse relationship between serum vitamin D status and body mass index (BMI) in studies of both diabetic (*r* = −0.173, 95% = −0.241 to −0.103, *p* = 0.000) and non-diabetic (*r* = −0.152, 95% = −0.187 to −0.116, *p* = 0.000) subjects. The evidence of publication bias was not found in this meta-analysis. In conclusion, the deficiency of vitamin D is associated with an increased level of BMI in the studies of both diabetic and non-diabetic subjects. Reliable evidence from well-designed future randomized controlled trials is required to confirm the findings from observational studies and to find out the potential regulatory effects of vitamin D supplementation to lower BMI.

## 1. Introduction

Obesity has been prevalent all over the world in association with a rise in type 2 diabetes and hypovitaminosis D over the last three decades [1]. If current trends continue, over one billion adults will be affected by obesity, and 2.7 billion will be overweight by 2025, as estimated by the World Obesity Federation. Due to the relationship of obesity to chronic diseases like some cancers, cardiovascular diseases, and type 2 diabetes (T2D), the disease is gaining in much importance [2]. Recently, vitamin D deficiency has attracted attention in the development of obesity. It is evident that circulating vitamin D deficiency is related with obesity and type 2 diabetes [1], and both hypovitaminosis D and obesity ends up in common diseases like T2D, cardiovascular disease, and some cancers [3].

The high vitamin D deficiency worldwide is usually a result of reduced cutaneous production due to decreased sun exposure and a reduced intake of vitamin D, which is related to increased mortality rates [4]. In the areas where the sun does not shine so often, vitamin D is not photosynthesized properly [5], therefore the population of European countries are usually deficient in vitamin D [6]. The deficiency of vitamin D is seen even in parts of the world that are considered as deserts and have long day lengths. It is well known that vitamin D is involved in bone mineralization, however, recent studies have shown that hypovitaminosis D is responsible for more pathologic conditions than previously thought. For example, Metabolic Syndrome (MS), Insulin Resistance, and Diabetes Mellitus could potentially develop as a result of a deficiency in vitamin D [7,8,9].

After binding to the vitamin D responsive elements in the promoter region, the vitamin D receptor (VDR), a nuclear transcriptional regulator, up or down regulates the gene transcription [10]. The biologically active form of vitamin D (1, 25-hydroxy vitamin D) plays a central role in a large variety of metabolic pathways by binding to the vitamin D receptor (VDR). The vitamin D receptor is expressed in a variety of cells, e.g., pancreatic beta cells of Langerhans, liver, adipose tissues, and muscle cells [11,12,13]. The 25 hydroxy vitamin D is usually considered as the biomarker for vitamin D status, however, well-defined cutoff values have not yet been established that parallel the optimal physiological vitamin D requirements. Therefore, there is a need to evaluate the vitamin D recommendations.

Consistent with our hypothesis that vitamin D deficiency and obesity are related, areas where there is a deficiency of vitamin D are also prevalent with obesity [14]. Whether it is the deficiency of vitamin D which leads to obesity or the obesity that leads to the deficiency of vitamin D is debatable. Scientists have suggested that a higher body mass index BMI can lead to lower vitamin D, as vitamin D, being a fat soluble hormone, is sequestered in the adipose tissues and only small quantities are consequently available for circulation [15]. Moreover, the production of enzymes (25-Hydroxylase and 1-hydroxylase) catalyzing the hydroxylation of vitamin D to its active form, is very low in obese individuals when compared to their lean counterparts [16], therefore the active metabolites of vitamin D (25-hydroxy vitamin D and 1, 25-hydroxy vitamin D) are formed in relatively less quantities in obese individuals.

We examined the prospective association between the levels of vitamin D and BMI. Sun exposure also affects the vitamin D status, therefore this association may be affected by the latitude of residency. In addition, different cultures and customs, method of determining vitamin D, developmental status of the country, and gender may have an effect on this correlation. We performed meta-regression to find out if these parameters had an influence on the association between vitamin D status and obesity.

## 2. Materials and Methods

We searched three databases, PubMed, Cochrane, and Embase, for the collection of the observational studies. The articles were collected from inception until March 2018. The following key words were used “25 (OH) D”, “25 (OH) vitamin D”, “vitamin D”, “Cholecalciferol”, “vitamin D3”, in combination with “BMI”, “Body mass index”, “obesity”, “waist circumference”, “abdominal obesity”, “overweight”, “adiposity”, “body size”, and “type 2 diabetes”, “T2D”, “fasting plasma glucose”, “HBA1C”, “Homeostasis model assessment of insulin resistance”, and “fasting plasma insulin”. The keywords were searched both as free terms and in combination with MeSH (medical subject headings) in PubMed and EMTREE in EMBASE. Observational studies were selected that showed the association of body mass index and 25-hydroxy vitamin D levels in the blood. We specified that every study must have been conducted on human beings, and the age of the individuals should be more than 18 years. We only included studies that were written in English. If the studies were commentaries, editorials, or case reports, they were excluded. We also hand searched the related articles for more references. The authors of the identified articles were contacted if the articles lacked essential information on the estimates and the moderators.

### Statistical Analysis and Outcome Measures

The correlation coefficient was used as a summary measure for the outcome studies. We gathered the results from all over the world, from different ethnicities and regions, and we assumed that the biological effect of vitamin D was different in different populations. Therefore, we followed the random effects model to compute the meta-analysis results. We used *I*^2^ as a test of consistency, which describes the total heterogeneity between the studies in the form of percentage, and *τ*^2^ was used as an estimate of reliability. The quality of the evidence was assessed by GRADE (Grades of Recommendation Assessment Development and Evaluation). Evidence derived from observational studies obtained a low initial GRADE score when compared to the randomized control trials which achieved a high initial GRADE score. A number of factors can upgrade or low grade these scores, e.g., inconsistency (unexplained heterogeneity between the studies), indirectness (compromised generalizability of results), imprecision (too long confidence intervals), and publication bias (small number of participants). The software used was comprehensive meta-analysis version 3 (Biostat, Inc., Englewood, NJ, USA), tools were used to perform the meta-analysis and the risk of bias assessment was done using Review Manager 5.3. Forest plot graphs were used to present the results and meta-regression was performed to evaluate the potential sources of bias.

## 3. Results

**Figure 1:**
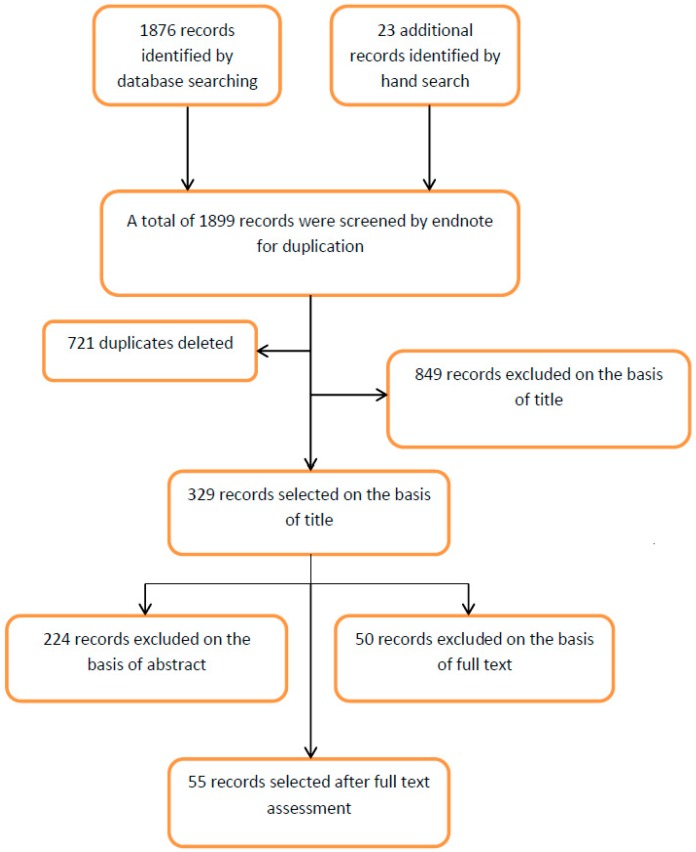
Flow sheet diagram for the study selection process.

A total of 1876 observational studies were retrieved electronically from Embase, PubMed, and Medline, and 23 observational studies were identified by other sources. Endnote software was used to screen the references for duplication and 721 references were deleted. Three hundred and twenty nine articles were selected for abstract assessment on the basis of titles. After abstract assessment, we selected 105 studies for further evaluation by full text assessment. Fifty five studies that reported the association between BMI and vitamin D status both in healthy adults and type 2 diabetes patient populations all over the world were finally selected for meta-analysis (Figure 1).

### 3.1. Included Studies

Participants from all of the 55 included studies were at least 18 years of age and belonged to diverse backgrounds from all over the word. Forty one studies were from Asia, thirteen from Europe, nine from America, and one from Australia. For the determination of vitamin D, twenty two studies used the chemiluminescence (CLIA) method, fifteen studies used the radioimmunoassay (RIA), nine studies used the enzyme linked immunosorbent assay (ELISA), eight studies used the electrochemiluminescence (ECLIA) method, five studies used the enzyme immunoassay (EIA), three studies used liquid chromatography-mass spectrometry (LC-MS), and two studies used high pressure liquid chromatography (HPLC). We performed random effects meta-analysis due to the huge diversity in the study population and the method of determination of vitamin D. We additionally estimated meta-regression to determine the effect of different parameters, e.g., the method of vitamin D determination, latitude, and different BMI groups, which were likely to confound the correlation between vitamin D status and BMI, ultimately affecting the overall effect size.

#### 3.1.1. Meta-Analysis of the Association of Vitamin D and BMI in Non-Diabetics

**Figure 2:**
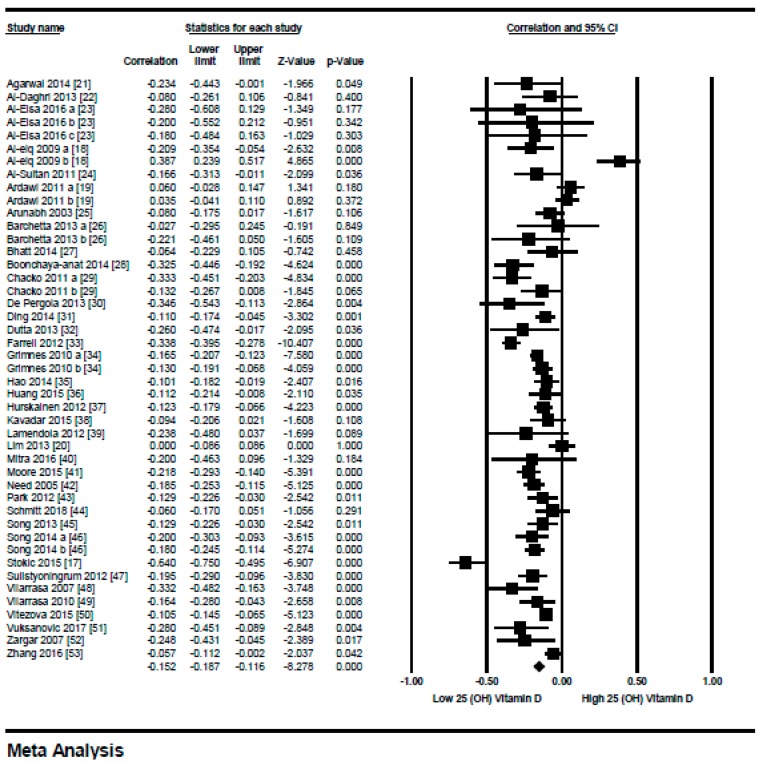
Non-diabetic subject studies: Forest plot shows the correlation between vitamin D status and BMI [18,19,20,21,22,23,24,25,26,27,28,29,30,31,32,33,34,35,36,37,38,39,40,41,42,43,44,45,46,47,48,49,50,51,52,53]. A random effects model was used to calculate the correlations and 95% confidence interval (CI) (*I*^2^ = 80.524%, *p* = 0.000).

We included 45 studies in this meta-analysis. The relationship between serum vitamin D status and BMI in non-diabetic subjects all over the world is shown in Figure 2. An overall inverse relationship (*r* = −0.152, 95% = −0.187 to −0.116, *p* = 0.000) (Figure 2) was seen between vitamin D status and BMI in this meta-analysis. All of the studies had a correlation coefficient between 0 to −0.5, except for one study from Novi Sad, Serbia [17], which showed a relatively stronger correlation (*r* = −0.64), other studies from Saudi Arabia [18,19] that showed a positive correlation, and a study from Korea [20] showed no relationship at all. Interestingly, all the studies that showed a positive correlation were from Saudi Arabia and the study population was female.

**Figure 3:**
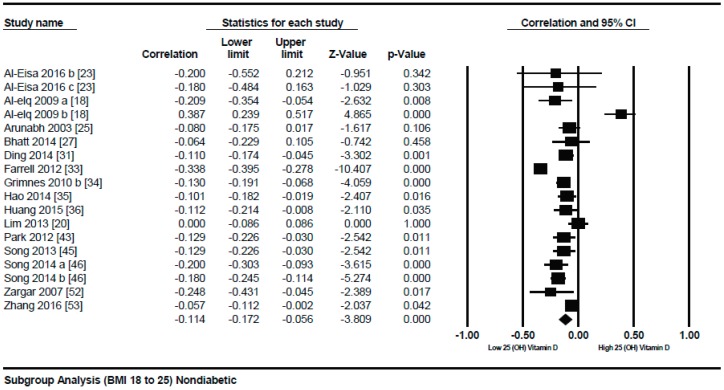
Non-diabetic subject studies for the lowest BMI quartile (18–25): Forest plot shows the correlation between vitamin D status and body mass index (BMI) [18,20,23,25,27,31,33,34,35,36,43,45,46,52,53]. A random effect model was used to calculate the correlations and 95% CI (*I*^2^ = 84.372%, *p* = 0.000).

**Figure 4:**
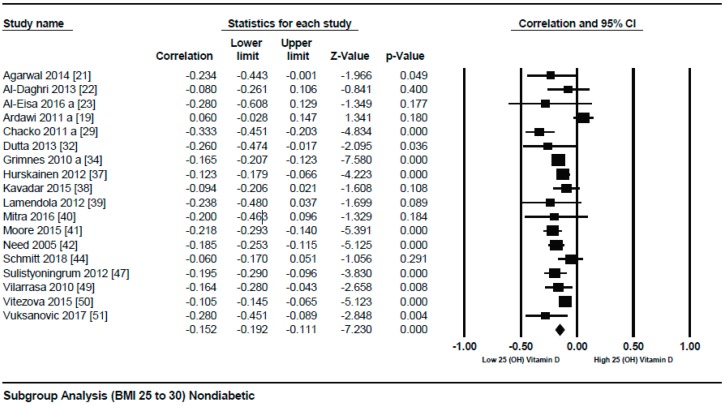
Non-diabetic subject studies for the medium BMI quartile (25–30): Forest plot shows the correlation between vitamin D status and BMI [19,21,22,23,29,32,34,37,38,39,40,41,42,44,47,49,50,51]. A random effect model was used to calculate the correlations and 95% CI (*I*^2^ = 64.411%, *p* = 0.000).

**Figure 5:**
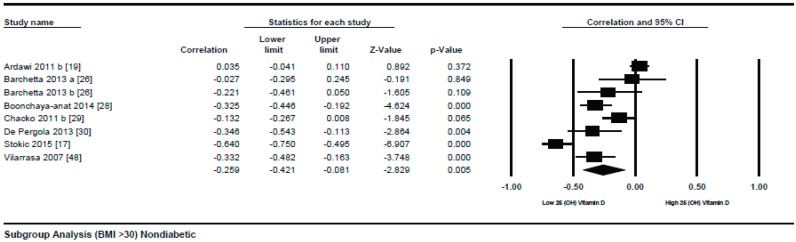
Non-diabetic subject studies for the highest BMI quartile (>30): Forest plot shows the correlation between vitamin D status and BMI [17,19,26,28,29,30,48]. A random effect model was used to calculate the correlations and 95% CI (*I*^2^ = 89.764%, *p* = 0.000).

A subgroup analysis according to the different quartiles of BMI showed a gradual strong correlation from the lowest to highest quartile. The correlation (*r* = −0.114, 95% = −0.172 to −0.056, *p* = 0.000) (Figure 3) was more attenuative in the lowest quartile of BMI (BMI = 18–25), however, it became stronger (*r* = −0.152, 95% = −0.192 to −0.111, *p* = 0.000) (Figure 4) in the second quartile of BMI (BMI = 25–30), and was strongest (*r* = −0.259, 95% = −0.421 to −0.081, *p* = 0.000) (Figure 5) in the third quartile of BMI (BMI ≥ 30).

#### 3.1.2. Meta Regression Analysis

**Figure 6:**
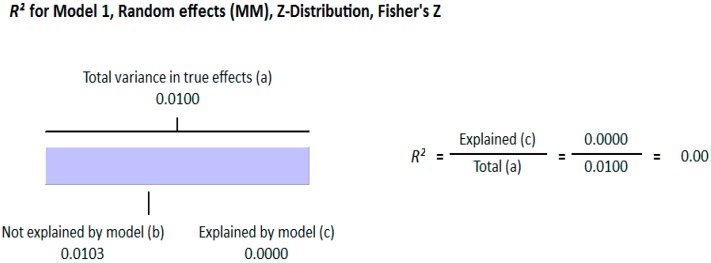
Non-diabetic subject studies. Regression analysis for the moderator “latitude”: *R*-squared representation shows the contribution of latitude on the heterogeneity of the results.

**Figure 7:**
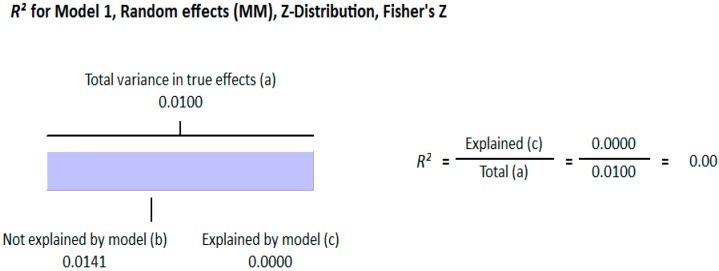
Non-diabetic subject studies. Regression analysis for the moderator “Method of vitamin D determination”: *R*-squared representation shows the contribution of method of determination of vitamin D on the heterogeneity of the results.

**Figure 8:**
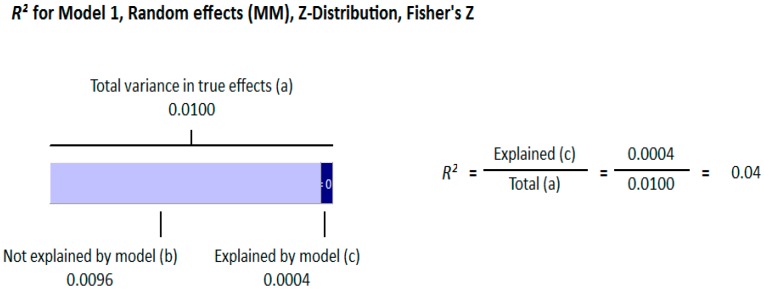
Non-diabetic subject studies. Regression analysis for the moderator “Different quartiles of BMI”: *R*-squared representation shows the contribution of different quartiles of BMI on the heterogeneity of the results.

**Figure 9:**
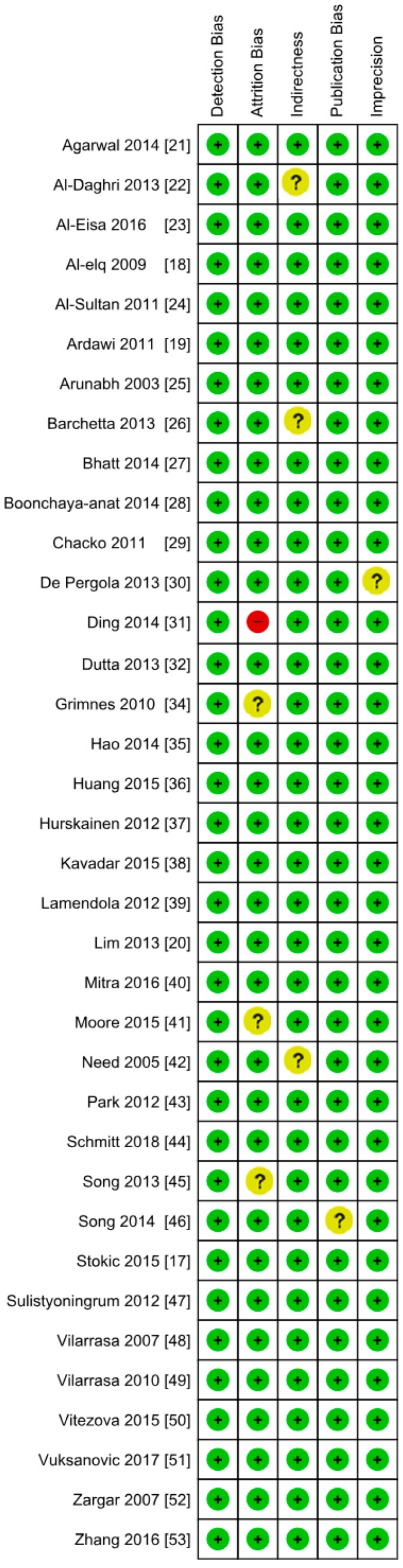
Authors’ assessment of the risk of bias in non-diabetic subject studies: Data shown for each individual study (the plus sign indicates a low risk of bias, the minus sign shows a high risk of bias, and the question mark indicates that the risk of bias is unknown) [17,18,19,20,21,22,23,24,25,26,27,28,29,30,31,32,34,35,36,37,38,39,40,41,42,43,44,45,46,47,48,49,50,51,52,53].

**Figure 10:**
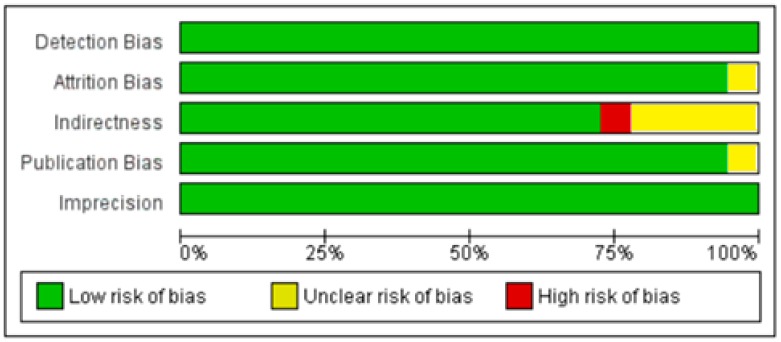
Author’s assessment of the risk of bias in non-diabetic subject studies: Data shown in percentages for all studies.

The meta-regression analysis of the effect of moderators on this correlation was also observed. The selected moderators were latitude, method of vitamin D determination, and different quartiles of BMI (18–25, 25–30, and >30). We did not find any effect of the moderator’s latitude (*R*^2^ = 0.000%, *p* = 0.000) (Figure 6) and method of vitamin D determination (*R*^2^ = 0.000%, *p* = 0.000) (Figure 7) on the relationship between vitamin D status and BMI (Figure 6 and Figure 7). However, 4% of heterogeneity between the relationship was due to the moderator “different quartiles of BMI” (*R*^2^ = 0.04%, *p* = 0.000) (Figure 8). The GRADE assessment for this meta-analysis is shown in Figure 9 and Figure 10.

##### Meta-Analysis of the Association of Vitamin D and BMI in Diabetics

**Figure 11:**
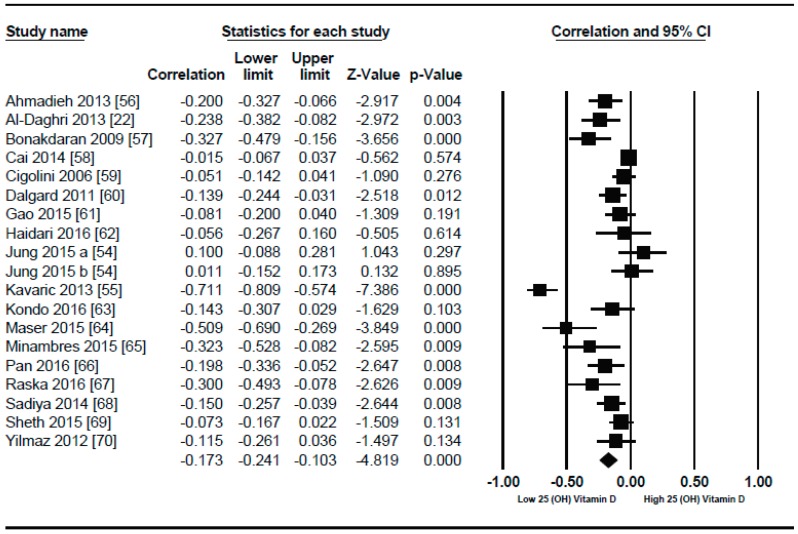
Diabetic subject studies: Forest plot shows the correlation between vitamin D status and BMI [22,54,55,56,57,58,59,60,61,62,63,64,65,66,67,68,69,70]. A random effect model was used to calculate the correlations and 95% CI (*I*^2^ = 80.520%, *p* = 0.000).

Nineteen studies were selected in this meta-analysis. In similar non-diabetic subject studies, the vitamin D status also had an inverse correlation (*r* = −0.173, 95% = −0.241 to −0.103, *p* = 0.000) (Figure 11) with BMI in diabetic subjects, showing a more pronounced relationship comparably. Some unusual relationships from this meta-analysis included a positive correlation between serum vitamin D levels and BMI.

**Figure 12:**
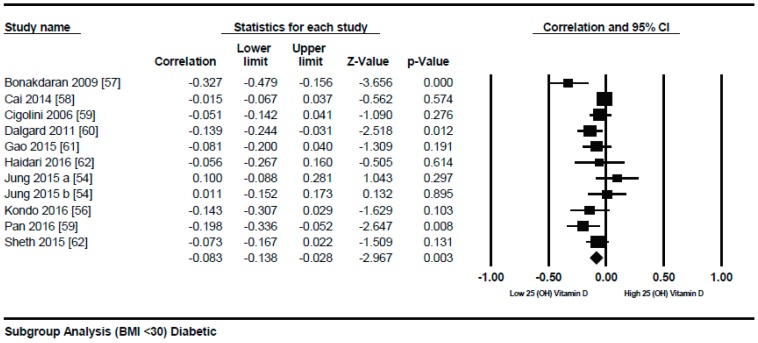
Diabetic subject studies for the lower BMI quartile (<30): Forest plot shows the correlation between vitamin D status and BMI [54,56,57,58,59,60,61,62]. A random effect model was used to calculate the correlations and 95% CI (*I*^2^ = 54.584%, *p* = 0.00).

**Figure 13:**
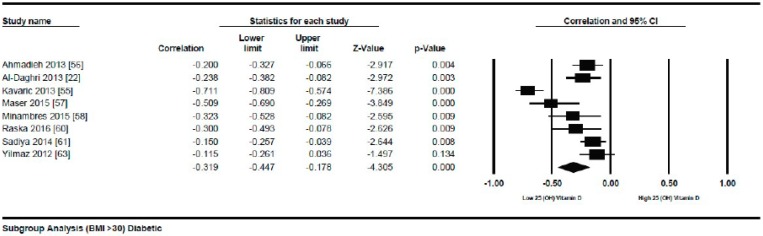
Diabetic subject studies for higher BMI quartile (>30): Forest plot shows the correlation between vitamin D status and BMI [22,56,57,58,60,61,63]. Random effect model was used to calculate the correlations and 95% CI (*I*^2^ = 82.465%, *p* = 0.00).

In both genders from Korea [54] and a study from Serbia [55] showed strong inverse correlation (*r* = −0.711). As described by τ^2^ (τ^2^ = 0.01), the between study variability was low. In diabetic patients, the *p* = 0.000) (Figure 12) with serum vitamin D levels when compared to the lower BMI quartile (BMI ≤ 30) (*r* = −0.083, 95% = −0.138 to −0.028, *p* = 0.000) (Figure 13) when a subgroup-analysis was performed.

##### Meta Regression Analysis

**Figure 14:**
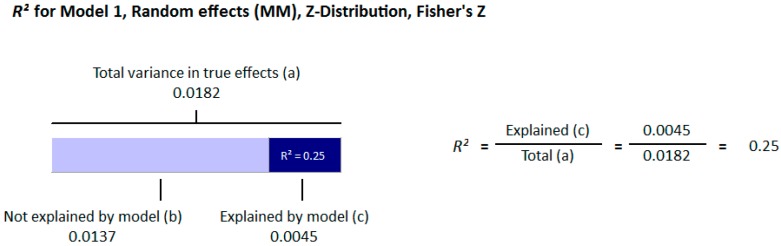
Diabetic subject studies. Regression analysis for the moderator “Different quartiles of BMI”: *R*-squared representation shows the contribution of different quartiles of BMI on the heterogeneity of the results.

**Figure 15:**
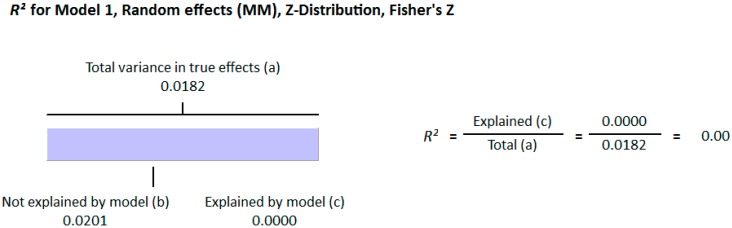
Diabetic subject studies. Regression analysis for the moderator “Latitude”: *R*-squared representation shows the contribution of latitude on the heterogeneity of the results.

**Figure 16:**
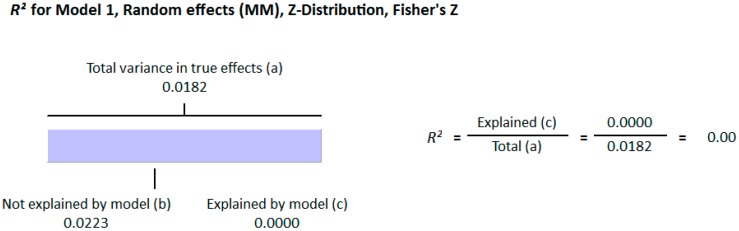
Diabetic subject studies. Regression analysis for the moderator “Method of vitamin D determination”: *R*-squared representation shows the contribution of the method of vitamin D determination on the heterogeneity of the results.

**Figure 17:**
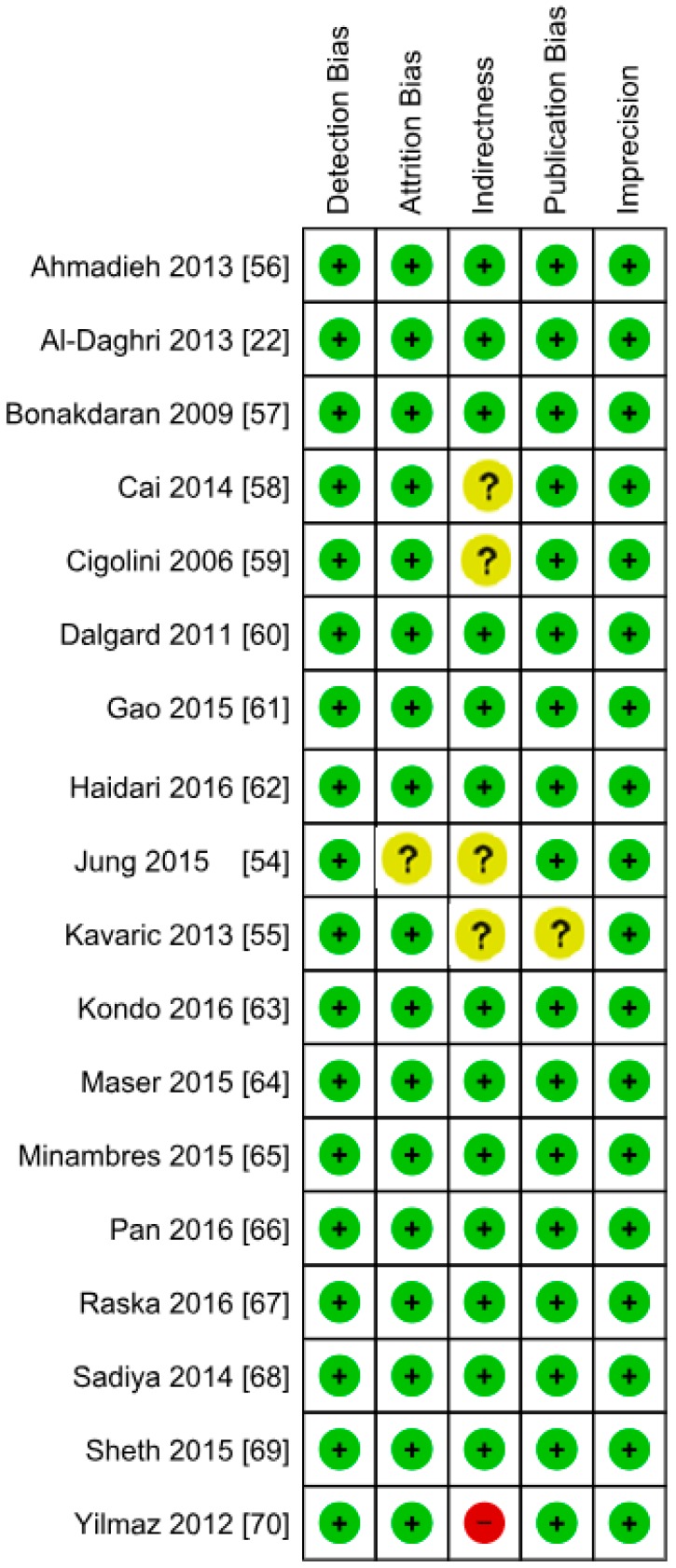
Authors’ assessment of the risk of bias in the diabetic subject studies: Data shown for each individual study (the plus sign shows a low risk of bias, the minus sign shows a high risk of bias, and the question mark indicates that the risk of bias is unknown) [22,54,55,56,57,58,59,60,61,62,63,64,65,66,67,68,69,70].

**Figure 18:**
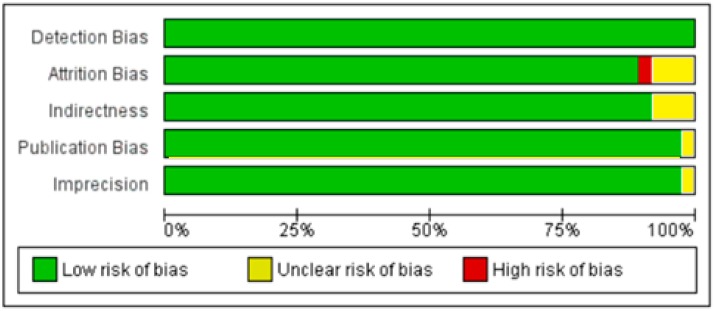
Authors’ assessment of the risk of bias in diabetic subject studies: Data shown in percentages for all studies.

The moderators selected for the meta-regression analysis were latitude, method of vitamin D determination and various categories of BMI. The *R*^2^ Graphics showed that 25% of heterogeneity was because of the different quartiles of BMI (*R*^2^ = 0.25, *p* = 0.00) (Figure 14), however latitude (*R*^2^ = 0.000%, *p* = 0.000) (Figure 15) and method of vitamin D determination (*R*^2^ = 0.000%, *p* = 0.000) (Figure 16) did not have an effect on the correlation of serum vitamin D levels and BMI. The GRADE assessment is shown in the Figure 17 and Figure 18 for this meta-analysis.

### 3.2. Excluded Studies

Sixteen studies [71,72,73,74,75,76,77,78,79,80,81,82,83,84,85,86] were excluded as the data were not harmonious to calculate the effect size in the form of correlation. Five studies [87,88,89,90,91] were disqualified because of their study design, which was not well-suited with the study in progress. Twelve studies [92,93,94,95,96,97,98,99,100,101,102,103] were omitted since they considered a mixed population (Diabetic and non-diabetic) for the determination of effect size.

## 4. Discussion

This review provides evidence that serum vitamin D status has an inverse relationship with BMI in diabetic and non-diabetic subjects. An overall weak correlation was seen in the non-diabetic subject population (*r* = −0.152, 95% = −0.187 to −0.116, *p* = 0.000) (Figure 2) when compared to the diabetic ones (*r* = −0.173, 95% = −0.241 to −0.103, *p* = 0.000) (Figure 11). When we performed the subgroup analysis according to different quartiles of BMI in non-diabetic subject studies, a gradual increase in the strength of correlation was seen with increasing BMI quartiles. However, an abrupt rise in the correlation was seen in the higher quartile of BMI when compared to the lower one in the diabetic subject studies.

In the lower quartile of BMI for the diabetic patient studies, a weak correlation (*r* = −0.083, 95% = −0.138 to −0.028, *p* = 0.000) (Figure 12) was seen, nearly one fourth of the higher BMI quartile of the diabetic subject studies (*r* = −0.319, 95% = −0.477 to −0.178, *p* = 0.000) (Figure 13). 

The strong inverse correlation between vitamin D deficiency and BMI might be due to the relationship of hypovitaminosis D and obesity to a number of diseases [104]. The coexistence of these two factors may have relevance to the development of some disease conditions, for example, type 2 diabetes is strongly related with obesity and vitamin D deficiency [1,15]. It has been observed previously that the synergistic effect of obesity and vitamin D deficiency can develop insulin resistance [1]. A study on animals also supports the hypothesis that vitamin D receptors and vitamin D can have a role in type 2 diabetes and obesity [105], and vitamin D receptors induced by 1, 25-(OH) vitamin D are more expressed in the adipose tissues in the obese when compared to lean subjects. The body mass index relates independently with hypovitaminosis D, and a decrease of 1.3 nM/L of vitamin D can add 1 kg/m^2^ of BMI [106].

The strong correlation (*r* = −0.319, 95% = −0.477 to −0.178, *p* = 0.000) (Figure 13) in the current study between BMI and serum vitamin D status in the higher BMI quartile is an indication that hypovitaminosis D could be the reason behind obesity and type 2 diabetes. However, the weak correlation (*r* = −0.083, 95% = −0.138 to −0.028, *p* = 0.000) (Figure 12) in the lower BMI quartile of type 2 diabetes patients indicated the existence of type 2 diabetes due to reasons other than hypovitaminosis D. The meta-regression studies in the current project also confirmed these results as we found that 25% (*R*^2^ = 0.25, *p* = 0.00) (Figure 14) of the variability in the correlation was due to BMI in diabetic subject studies and that 4% (*R*^2^ = 0.04%, *p* = 0.000) (Figure 6) of the variability in the correlation was because of BMI in the non-diabetic subject studies.

There was a parallel increase in vitamin D deficiency and BMI over time. Exposure to sunlight is a huge natural source of vitamin D. The UV-B radiations from sunlight are responsible for the synthesis of vitamin D in the body. With industrialization, these radiations have been reduced in many ways, e.g., they are absorbed and scattered by the concrete ground and buildings [107], the gases produced by vehicles and industry like NO_2_, SO_2_, and O_3_ from ozone are absorbed in the UV-B region of the spectrum, while carbon pollution also affects the irradiance of UV-B [108]. The new trends of the consumption of low fat milk instead of whole milk may also be a culprit in the critical reduction in vitamin D status. The biologically active metabolite of vitamin D is 1, 25-(OH) vitamin D, which is produced by the hydroxylation of 25-(OH) vitamin D. This biologically active form of vitamin D has the ability to interact with vitamin D receptors.

Although latitude can explain the spread of hypovitaminosis D geographically, as most of the vitamin D needed is prepared photosynthetically, many geographical trends can overrule these effects. The diet of the population, their clothing style, their occupation, means of leisure and travel, time of sun exposure, urbanization, and skin pigmentation can have an impact on the photo production of vitamin D, therefore people may have vitamin D deficiency even at very low latitudes [109,110]. Populations even from sunny climates are deficient in vitamin D, thus, living in sunny areas does not assure protection against vitamin D deficiency. There existed a general inverse relationship between vitamin D deficiency and obesity in both studies on diabetic and non-diabetic subjects in the current meta-analysis, except for some studies on the female population from the KSA (Kingdom of Saudi Arabia), showed positive relationships in non-diabetic subject studies [18,19]. The female population in the KSA is usually entirely covered with black gowns; we assumed that after years of being covered, the female population in KSA might adapted some mechanisms that compensate for the adverse effect of vitamin D deficiency against obesity. This was in line with our previous observations where people of African origin with darker skin shades showed an unusually positive relationship between serum vitamin D levels and plasma glucose status [15]. These observations strengthen the idea of the development of a recovery mechanism after stress is imposed.

The relationship of adiposity and hypovitaminosis D is debatable. The inverse relationship could be due to the restoration of vitamin D in the adipose tissues, being a fat soluble vitamin, or it may be due to the reason that vitamin D may regulate the adipose tissue so that it can contribute to more fat mass and obesity related complications [111]. Some scientists have suggested that there may be a dilution effect due to the large body volumes for reduced circulating vitamin D [112]. Others have found an association with season and a three month exposure to sunlight before sampling was found to have strong correlation. The existence of a dilution factor typically explains the presence of low vitamin D levels in obese individuals. It is therefore suggested that individuals with a BMI higher than 30 should take vitamin D more frequently and at higher doses [113]. There may be a possibility that the skin of the obese person is not able to prepare vitamin D properly, like the skin of older individuals. The inverse correlation between vitamin D and circulating lipids has also been seen in children and adolescents, however, the extent of this correlation depends on the exposure to UV radiation [114].

These studies have shown that vitamin D may regulate the immune cell derived and adipose tissue related inflammations.

A recent study carried out on 9649 adults reported that the inverse correlation between CRP’s (C-reactive proteins) and serum vitamin D status may relate to obesity related complications. The reason behind this relationship is still unknown, however, the supplementation of 25-(OH) vitamin D can alleviate obesity related metabolic complications and inflammations [115,116,117,118]. Further studies are required to find out the mechanism underlying vitamin D related obesity and its complications.

The non-alcoholic fatty liver disease related factors such as diabetes are associated with vitamin D deficiency, therefore, it is considered that vitamin D may have a role in the development of steatosis [119]. Numerous signals and pathways are responsible for energy homeostasis and consequently, body weight. These circulating signals may include insulin and pancreatic polypeptides from the pancreas and may include leptin and resistin from adipose tissue. Vitamin D could be the part of the central control mechanism that regulates the body mass index through homeostatic pathways [13,120]. The expression of many genes related to vitamin D metabolism is also involved in the alteration of body mass index (BMI). BMI related genome wide association studies have identified more than two hundred and fifty variants [121].

One of the strengths of our study was that we used a systematic search strategy for the collection of observational studies. The quality of the studies was determined by using the “Grading of Recommendations Assessment, Development, and Evaluation (GRADE)”. The 95% confidence intervals were not too wide in both the diabetic and non-diabetic subject studies, which relates to the clinical importance of vitamin D to BMI. Although the number of participants in the observational studies was high, there are chances of residual confounding, which is the limitation of this study. The confounding could be due to differences in the population like different age groups. Subjects with different age groups have different exposure times to the sun and different rates of vitamin D synthesis due to differences in the capacity of the skin to synthesize vitamin D. In contrast to randomized control trials (RCTs), the observational studies had the disadvantage of not being randomized and blinded, which can lead to heterogeneity. Furthermore, we did not obtain the exact information related to the exposure to sunlight and vitamin D intake which can confound the results. The evidence was considered to be of moderate quality depending on all the strengths and weaknesses of the study.

## 5. Conclusions

The current meta-analysis demonstrated an inverse relationship between vitamin D status and BMI in both the diabetic and non-diabetic subjects, however, this association was more pronounced in the diabetic patients. The correlation was directly related to the BMI quartiles and the highest BMI quartile had the strongest correlations in both the diabetic and non-diabetic populations. In the subgroup analysis of the diabetic subject studies, an abrupt increase in the correlation in the higher quartile showed the positive relationship of hypovitaminosis D, obesity, and type 2 diabetes. The meta regression analysis also confirmed this positive relationship (*R*^2^ = 0.25, *p* = 0.00) (Figure 14).
